# Transcriptome analysis of *Phytophthora litchii* reveals pathogenicity arsenals and confirms taxonomic status

**DOI:** 10.1371/journal.pone.0178245

**Published:** 2017-06-01

**Authors:** Jinhua Sun, Zhaoyin Gao, Xinchun Zhang, Xiaoxiao Zou, Lulu Cao, Jiabao Wang

**Affiliations:** 1 The Environment and Plant Protection Institute, Chinese Academy of Tropical Agricultural Sciences, Haikou, PR China; 2 The Institute of Tropical Bioscience and Biotechnology, Chinese Academy of Tropical Agricultural Sciences, Haikou, PR China; US Department of Agriculture, UNITED STATES

## Abstract

Litchi downy blight, caused by *Peronophythora litchii*, is one of the major diseases of litchi and has caused severe economic losses. *P*. *litchii* has the unique ability to produce downy mildew like sporangiophores under artificial culture. The pathogen had been placed in a new family Peronophytophthoraceae by some authors. In this study, the whole transcriptome of *P*. *litchii* from mycelia, sporangia, and zoospores was sequenced for the first time. A set of 23637 transcripts with an average length of 1284 bp was assembled. Using six open reading frame (ORF) predictors, 19267 representative ORFs were identified and were annotated by searching against several public databases. There were 4666 conserved gene families and various sets of lineage-specific genes among *P*. *litchii* and other four closely related oomycetes. In silico analyses revealed 490 pathogen-related proteins including 128 RXLR and 22 CRN effector candidates. Based on the phylogenetic analysis of 164 single copy orthologs from 22 species, it is validated that *P*. *litchii* is in the genus *Phytophthora*. Our work provides valuable data to elucidate the pathogenicity basis and ascertain the taxonomic status of *P*. *litchii*.

## Introduction

Litchi (*Litchi chinensis Sonn*) is a delicious subtropical fruit in Southern China, Thailand, India, Vietnam, Australia, and South Africa. Litchi downy blight, caused by *Peronophythora litchii* Chen ex Ko et al., is one of the major diseases of litchi[1]. A previous study has reported that *P*. *litchii* exclusively infects litchi, but later research has indicated that it can also infect the fruits of longan, tomato, and pawpaw upon artificial inoculation[1–7]. The causal agent infects new shoots, young leaves, flowers, panicles, and fruits[6]. Diseased tissues turn brown and become covered with masses of white sporangia and sporangiophores. The pathogen further causes fruit rot and results in severe post-harvest losses (up to 60%)[8].

*P*. *litchii* is a hemi-biotrophic plant pathogen, which relies on living host cells at the early stage of infection and switches to a destructive necrotrophic lifestyle later. Similar to other oomycete pathogens, it often releases zoospores from sporangia under wet conditions[9]. Sporangia and zoospores of *P*. *litchii* attach to the hosts by swimming with motile flagella or dispersing through splash droplets. Upon docking at potential infection sites, zoospores stick onto host surfaces through adhesive glycoproteins and germinate[9]. The germinated zoospores penetrate the plant surface and invade host tissue by extending a germ tube. Host colonization then occurs and ultimately leads to cell death and tissue collapse[9]. At the later stage of pathogen infection, sporangia are formed to initiate the next infection cycle. The polycyclic nature of the pathogen results in the dispersal of inoculum over an extended time and wide area.

*P*. *litchii* is an oomycete, which is a fungus-like diploid eukaryote but evolutionarily related to brown algae. Peronosporomycetidae, a subclass of oomycete, include Peronosporales, Pythiales, and Albuginales. Many plant diseases are classified under Peronosporales, such as *Phytophthora infestans* (causes the potato late blight) and *Hyaloperonospora arabidopsidis* (causes downy mildew of *Arabidopsis thaliana*)[10–12]. *P*. *litchii* was first isolated by Chen and was described in a new genus[2,3]. The causal agent produces differentiated sporangiophores with dichotomous branchlets. When the growth of sporangiophores was terminated, sporangia on each tip of branchlets enlarged and matured simultaneously. The determinate sporangiophore is characteristic of downy mildews. *P*. *litchii* resembles downy mildews in terms of sporangiophore morphology, while characteristics of the mycelium and sexual reproduction, as well as the fact that the species can be easily cultured, renders it similar to *Phytophthora* and *Pythium* species. Before the advent of molecular phylogenetic studies in oomycetes, the former had been placed in the Peronosporaceae, while the latter were placed in the Pythiaceae. Consequently, the family Peronophythoraceae had been introduced[3]. Soon afterwards, the indeterminate sporangiophore was reported by Chi *et al*. It was suggested that *P*. *litchii* should be transferred to *Phytophthora*[4]. Further studies have indicated that the sporangiophores of *P*. *litchii* are basically determinate and occasionally indeterminate. However, the traditional taxonomic status of *Peronophythora* is mainly based on its morphological and physiological characteristics. In recent years, DNA sequences have been used in the phylogenetic studies of *P*. *litchii*. Analysis of ribosomal 28S and ITS sequences have revealed that *P*. *litchii* should be included in *Phytophthora* because of its closer phylogenetic relationship to *Phytophthora* than to downy mildews[13–15]. After considering both morphological and genetic characteristics, Zhang *et al*. insisted that the taxonomic status of *P*. *litchii* as a distinct transitional species should be retained[16]. Most recently, the sequenced genome of *P*. *litchii* has provided convincing evidence that the *P*. *litchii* belongs to *Phytophthora*[17]. But there is still a debate about the relationship between *Phytophthora* and downy mildews[18].

There is an evolutionary arms race between host plants and their pathogens[19]. Pathogens have developed weaponry to facilitate penetration, colonization, nutrient acquisition, and host-defense response. Conversely, plants have established a system to recognize and defend against invading pathogens. Pathogen-associated molecular patterns (PAMP)-triggered immunity (PTI) mechanisms have evolved to restrict infection and growth of pathogens in plants[19,20]. However, successful pathogens can overcome PTI by producing various effectors. In response, plants have developed an additional defense system that recognize pathogen effectors and lead to effector-triggered immunity (ETI)[19]. ETI is a robust response that can result in programmed cell death (hypersensitive response, HR), which can kill both pathogen and pathogen-infected cells. As the cycle of subversion and recognition evolves, hosts and pathogens are modulated by the interplay between pathogen effectors and their host counterparts[19,21].

Recent molecular studies on oomycete pathogens have focused on secreted effectors, which are ubiquitous in pathogens and manipulate the defense responses of host plants[22,23]. The apoplastic and cytosolic effectors are discerned dependent on the site of action[22,24]. Apoplastic effectors, which are located at the pathogen-host interface, fulfill functions outside the host cell to disturb defense[22]. Apoplastic effectors include several groups, such as elicitins and elictin-like, NPP1-like proteins (necrosis-inducing *Phytophthora* protein), CBEL (cellulose binding, elicitor, and lectin-like) proteins, and protease inhibitors. Elicitins and elictin-like proteins harbor a 98-amino-acid domain, which include a core of six conserved cysteines in the C-terminal. These are a type of PAMPs and can trigger the PTI of hosts[22]. NPP1-like proteins can promote programmed cell death in dicotyledonous plants with an NPP1 domain[25,26]. Cytoplasmic effectors, which translocate into plant cells and interfere with the host physiological process, include several effector groups such as RXLR and Crinkle (crinkling and necrosis, CRN)[22]. RXLR-type effectors are named for an N-terminal RXLR-dEER amino acid motif, which assists in the translocation of proteins into the host cytoplasm with a signal peptide[27,28]. Meanwhile, about half of RXLR effectors display a conserved core α-helical fold (termed “WY-domain”) on C-terminal regions, which execute the actual effector activity[29–31]. These effectors suppress PTI or activate ETI depending on the host genotype[22,27,28]. In addition to RXLR effectors, the CRN protein family produces leaf crinkling and necrosis phenotype with a conserved “LXLFLAK” motif[10,32].

Despite its economic importance, molecular study on *P*. *litchii* has lagged far behind other oomycete species. High-throughput sequencing techniques pave the way to collect genetic data rapidly and cost effectively for functional studies in non-model species. To ascertain the taxonomic status and gain more insight into the molecular pathogenicity mechanisms of *P*. *litchii*, we report the Illumina sequencing, *de novo* assembly, annotation and analysis of the *P*. *litchii* transcriptome herein. We investigated the potential effector arsenal (including RXLR effectors) and confirmed the phylogenetic status of *P*. *litchii*. Overall, our results serve as a crucial foundation for further study.

## Materials and methods

### Oomycete materials

*P*. *litchii* strain was isolated from diseased fruit of litchi in Hainan, cultured routinely on 10% V8 agar media at 25°C in the dark. Two-week-old plates were flooded with 10 mL of sterile water and kept at 4°C for 2 hours to promote zoospores release. Mycelia, sporangia and zoospores were collected by centrifugation at 2,000 × *g* for 10 min and immediately preserved in liquid nitrogen for RNA isolation.

### RNA isolation, cDNA library preparation and Illumina sequencing

Total RNA was extracted using TRIzol reagent (Invitrogen, USA) according to the manufacturer’s instructions. The quality and quantity of RNA was checked by gel electrophoresis and spectrophotometry. Furthermore, Agilent 2100 Bioanalyzer (Agilent Technologies, Inc.) was applied to assess the integrity of the RNA sample. Prior to cDNA synthesis, RNA samples were treated with DNase I (Promega) to remove contaminating genomic DNA. The purified RNA was dissolved in RNase-free water and stored at -80℃ until used.

RNA-seq libraries were prepared following TruSeqTM RNA sample preparation Kit from Illumina (San Diego, CA), using 5 μg of total RNA. Shortly, mRNA was isolated with oligo (dT) magnetic beads and was fragmented into small pieces using fragmentation buffer. cDNA synthesis, end repair, A-base addition and ligation of the Illumina-indexed adaptors were performed according to Illumina’s protocol. The cDNA fragments of 300–500 bp on 2% Low Range Ultra Agarose were selected, then the products were enriched by PCR amplification using Phusion DNA polymerase (NEB) for 15 PCR cycles. After quantified by TBS380, cDNA library was sequenced using Illumina HiSeq^TM^ 2000 (2 × 100 bp read length). All the experiments were carried out in the Majorbio corporations (Shanghai, China).

### Transcriptome assembly and function annotation

The raw reads were cleaned by eliminating adapters, low-quality sequences and discarded reads with more than 10% of bases that had a q-value lower than 20. The filtered and trimmed reads were assembled *de novo* using Trinity program (v20140717) with the default settings[33]. To eliminate redundant sequences, transcripts were clustered based on sequence similarities, and the longest transcript in each cluster represented the final assembled genes that were subjected to function annotation. Open reading frames (ORFs) in the genes were predicted by TransDecoder (http://transdecoder.sourceforge.net/) with 180 bp set as the minimum ORF length. The longest ORF was extracted from each gene as the candidate protein sequence. The quality of the assembly was benchmarked against the core set of eukaryotic genes using the Core Eukaryotic Genes Mapping Approach (CEGMA) algorithm (with a cutoff e-value of 1e^-5^) and Benchmarking Universal Single-Copy Orthologs (BUSCO) referring to core eukaryote genes[34,35].

Assembled genes were compared to the following database: NCBI non-redundant protein sequences (Nr), SwissProt, Eukaryotic Orthologous Groups (KOG) and Kyoto Encyclopedia of Genes and Genomes (KEGG) using blastx with a cutoff e-value of 1e^−5^, NCBI non-redundant nucleotide sequences (Nt) using blastn with a cutoff e-value of 1e^-5^[36,37]. The candidate proteins were searched against Pfam database for conserved domains with hmmsearch[38,39]. Based on the results of the Nr and Pfam database annotation, Blast2GO was conducted to obtain Gene Ontology (GO) annotation[40].

### Identification of putative secreted proteins and pathogenesis-related effectors

The genes were translated to all possible ORFs that encoded at least 60 amino acids (aa) from both strands. SignalP was used to identify an amino terminal signal peptide[41]. The secretome of *P*. *litchii* consisted of the proteins with a D-score more than 0.45 and without predicted transmembrane (TM) domains. RXLR and CRN effectors were initially identified based on the function annotation. In a second approach, RXLR and CRN effectors were identified with hmmsearch using Hidden Markov Model (HMM) profile[10,32]. For RXLR effectors identification, a regex method was used by script. String searches for the motif (^\w{10,40}\w[1,96]R\wLR\w{1,40}[ED][ED][KR]) within the secretome were conducted[10], which allow for a signal peptide between residues 10–40, followed by the RXLR motif within the next 100 residues, followed by the EER motif, allowing D and K replacements to E and R, respectively[10]. To search for the conserved WY-domain, HMMER was used to search the C-terminal sequences of the predicted RXLR effectors using the previously described HMM models[10]. Hits with a positive HMM score were considered as a putative effector protein.

### Identification of orthologous genes and phylogenetic inference

Identification of single-copy orthologous genes are critical to comparative analysis. Here, we grouped the predicted proteins of *P*. *litchii* and other 21 oomycetes using blastp[36] (the sequences of *Albugo laibachii*, *Hyaloperonospora arabidopsidis*, *Phaeodactylum tricornutum*, *Phytophthora infestans*, *Phytophthora kernoviae*, *Phytophthora lateralis*, *Phytophthora nicotianae*, *Phytophthora parasitica*, *Phytophthora ramorum*, *Phytophthora sojae*, *Plasmopara halstedii*, *Pythium aphanidermatum*, *Pythium arrhenomanes*, *Pythium irregulare*, *Pythium iwayamai*, *Pythium ultimum*, *Pythium vexans*, *Saprolegnia declina*, *Saprolegnia parasitica* and *Thalassiosira pseudonana* obtained from the Ensembl Genomes database: ftp://ftp.ensemblgenomes.org/pub/protists/release-33/, and the sequences of *Phytophthora capsici* obtained from the DOE Joint Genome Institute (JGI) database: http://genome.jgi.doe.gov). The proteins of 22 oomycetes did self-blastp-self search with a cutoff e-value of 1e^-10^. The pairwise similarities were used to identify single-copy genes in each species with Markov cluster (MCL) algorithm at an inflation rate (I) of 2. The member of group with just one gene was identified as single-copy gene. On the other run, the predicted proteins of *P*. *litchii* were used as queries to search against other 21 species separately by reciprocal blast hit (RBH) approach. The best hits of each protein with a cutoff e-value of 1e^–6^ were retrieved. Secondly, the proteins of each 21 species were used as queries to search against *P*. *litchii* and the previous filters were carried out. The pair of genes was treated as orthologs if they met the “bidirectional best hit” law. Multiple orthologs-identify were conducted among the other 21 oomycetes. The orthologous group was ascertained if any two genes were orthologs among 22 species.

The single-copy orthologs were both single copy in each species and orthologs among all species. Each group of single-copy orthologs was aligned alone using MAFFT[42]. A concatenated alignment dataset was produced by putting each ortholog alignment in series by perl script. Phylogenetic analysis was carried out by using the RAxML program[43]. The PROTGAMMALGF model, which was the best model by using model-test program, was used to construct the phylogenetic tree with 1000 bootstrap trials. After consensus tree was constructed, the tree branch was estimated by proml program using phylip-3.696[44].

On the other hand, an all-against-all blastp (with cutoff e-value of 1e^-10^) was performed amongst predicted protein sequences of *P*. *litchii* and other four oomycete species: *H*. *arabidopsidis*, *P*. *halstedii*, *P*. *infestans*, and *P*. *sojae*[10,11,18,45]. The pairwise similarities served as input for clustering by the OrthoMCL approach with default settings[46]. The homologous proteins were grouped using Markov cluster (MCL) algorithm at an inflation rate (I) of 2.

## Results

### Illumina sequencing and sequence assembly

Illumina sequencing yielded about 52.5 million pair-end reads with an average length of 200 bp. After removing adaptor sequences, ambiguous reads, and low-quality reads, we obtained approximately 49.6 million cleaned high-quality reads (Table 1). Using the Trinity program, about 44.6 million cleaned reads were further assembled into 23637 transcripts (including isoforms) with an average length of 1284 bp and an N50 of 2176 bp (Table 1). The length of assembled transcripts ranged from 201 to 25551 bp. A set of 19267 unigenes (non-redundant transcripts) by selecting only the longest sequence among isoforms were used for subsequent analyses. More than half of the genes (10927, 56.7%) were longer than 500 bp, and 6538 (33.9%) genes were longer than 1 kb (S1 Fig). The GC content of assembly transcripts was about 51.83% (Table 1). Assembly completeness was evaluated using CEGMA and BUSCO pipeline. Totally, 241 of the 248 (97.2%) widely conserved core eukaryotic genes were mapped against the assembly of *P*. *litchii* transcriptome by CEGMA method. Among the 429 single-copy eukaryotic orthologous genes included in BUSCO analysis, there were 353 (82.3%) complete single-copy genes, 15 (3.5%) fragmented genes, and 61 (14.2%) genes missing in *P*. *litchii*.

**Table 1:** Summary of transcriptome sequencing and assembly for *P*. *litchii*.

Category	Count
Raw reads	52477156
Clean reads	49595837
Assembled reads	44613368
Transcripts	23637
Maximum length of transcripts (bp)	25511
Minimum length of transcripts (bp)	201
Mean length of transcripts (bp)	1284
Transcripts size N50 (bp)	2176
Unigenes	19267
Maximum length of unigenes (bp)	23217
Minimum length of unigenes (bp)	177
Mean length of unigenes (bp)	975
Unigenes size N50 (bp)	1533
GC content of genes (%)	51.83

### Gene function annotation

To elucidate potential gene functions, the gene annotation was carried out against Nt, Nr, KOG, KEGG, SwissProt, and Pfam database. A total of 17647 genes (91.6%) were successfully annotated in at least one database (Table 2), whereas the remaining may represent additional genes that were not represented in the annotated protein databases or sequences that were too short to produce hits (Table 2). There is a large number of hits which matched the sequences of *P*. *sojae* (51.6%) and *P*. *infestans* (37.0%) (S2 Fig).

**Table 2:** Overview of functional annotation of the *P*. *litchii* transcriptome.

Database	Number of annotated genes	Percentage (annotated/total number of genes, %)
Nr Annotation	17245	89.50
Nt Annotation	6417	33.30
SwissProt Annotation	9121	47.34
PFAM Annotation	11080	57.50
GO Annotation	12933	67.12
KOG Annotation	6920	35.91
KEGG Annotation	4624	23.99
Total annotated genes	17647	91.59

**Fig 1:**
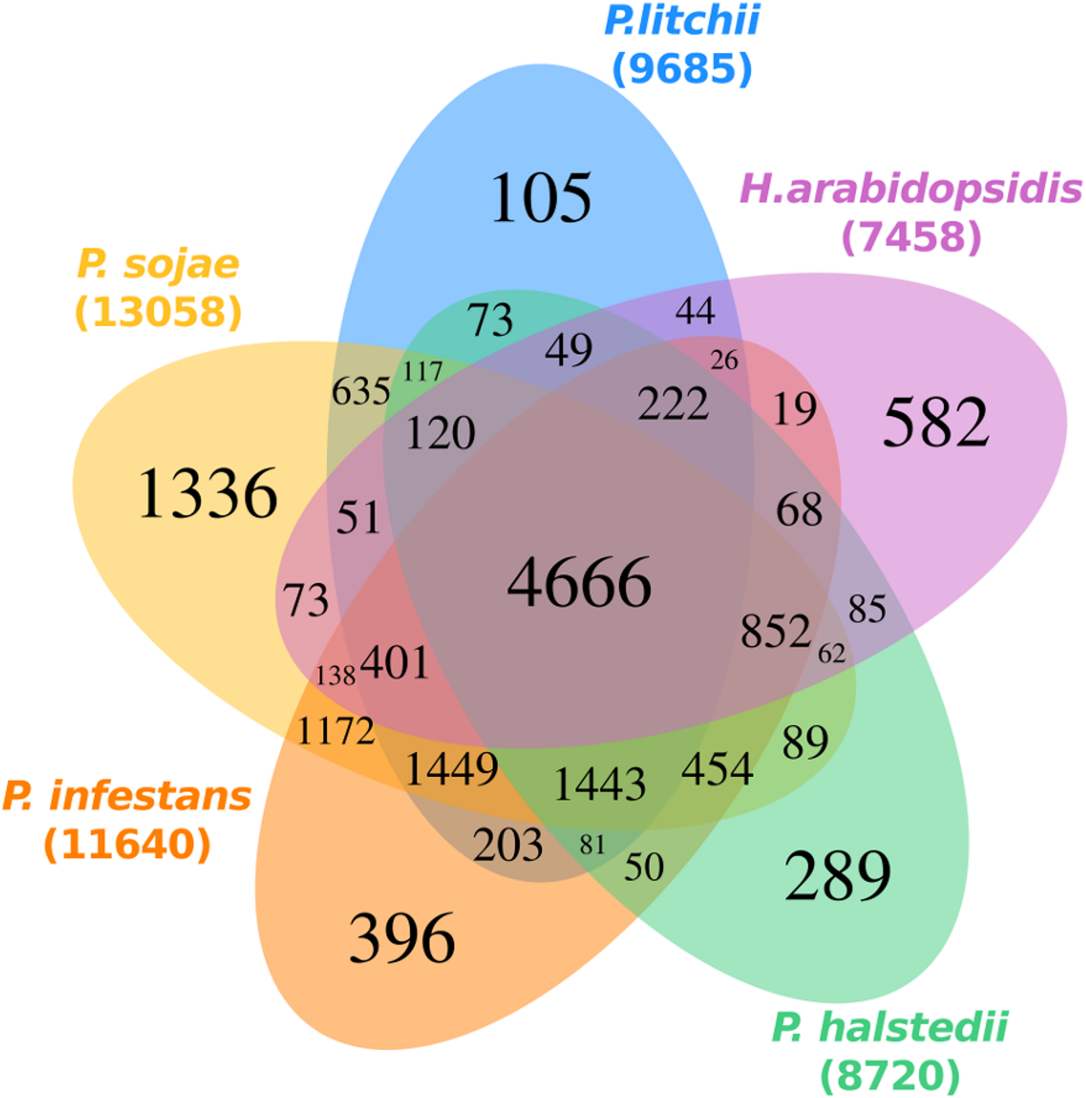
Five-way-Venn-diagram of unique and shared gene families among oomycete species. Homologous proteins in *P*. *litchii*, *H*. *arabidopsidis*, *P*. *halstedii*, *P*. *infestans*, and *P*. *sojae* were clustered into gene families using OrthoMCL. Numbers in each section refer to number of gene families (not genes). Overlapping regions denote groups with at least one protein of all species that are part of the intersection. The number under the organism name refer to the total number of clusters.

To better define conserved genes among *P*. *litchii* (19267), *H*. *arabidopsidis* (14321), *P*. *halstedii* (15469), *P*. *infestans* (17787), and *P*. *sojae* (26489), all protein from five species were clustered into 15350 groups (Fig 1). A total of 9685 orthologous groups for *P*. *litchii*, 7458 for *H*. *arabidopsidis*, 8720 for *P*. *halstedii*, 11640 for *P*. *infestans*, and 13058 for *P*. *sojae* were generated (Fig 1). Among them, 4666 orthologous groups were shared by all five species. A total of 635 gene families were shared only by *P*. *litchii* and *P*. *sojae*, whereas just 44 gene families were shared only by *P*. *litchii* and *H*. *arabidopsidis* (Fig 1). In addition, 105 gene families of *P*. *litchii*, including 257 proteins, had no orthologs in the other four oomycetes species (Fig 1).

Based on GO annotation, 12933 (67.12%) genes were assigned to 50 subcategories belonging to three major functional categories (biological process, cellular component, and molecular function) (S3 Fig). Among the categories of biological process, a significant proportion of clusters was assigned to “cellular process” (8384, 64.8%) and “metabolic process” (7772, 60.1%). In the categories of cellular component, the great majority of genes were related to the terms “cell” (6907, 53.4%) and “cell part” (6907, 53.4%). In the categories of molecular function, the most represented terms were “binding” (8369, 64.7%) and “catalytic activity” (6755, 52.2%) (S3 Fig).

To classify orthologous gene products, 6920 (35.9%) genes were functionally classified into 25 KOG categories (Table 2, S4 Fig). Among these groups, the cluster of “general function prediction only” (1078, 15.6%) represented the largest group, followed by “signal transduction mechanisms” (930, 13.4%), “posttranslational modification, protein turnover, chaperones” (844, 12.2%) and “translation, ribosomal structure and biogenesis” (473, 6.8%). The cluster of “cell motility” (12, 0.2%) and “extracellular structures” (21, 0.3%) represented the smallest groups (S4 Fig).

To identify the biological functions and interactions of genes, the genes were mapped to the reference canonical pathways in KEGG. A total of 4,624 (24.0%) genes were assigned to 32 groups, including 260 specific pathways (Table 2, S1 Table, S5 Fig). Major KEGG pathways can be divided into five categories: (i) cellular processes, (ii) environmental information processing, (iii) genetic information processing, (iv) metabolism, and (v) organismal systems (S5 Fig). Abundant genes (540, 11.7%) were involved in signal transduction (S1 Table, S5 Fig). Among them, some important pathways such as “MAPK signaling pathway” (126, 2.7%) and “calcium signaling pathway” (60, 1.3%) were identified. “T cell receptor signaling pathway” (61, 1.3%), “p53 signaling pathway” (25, 0.5%), “plant hormone signal transduction” (19, 0.4%), and “notch signaling pathway” (16, 0.3%) potentially involved in pathogenesis, were also identified (S1 Table, S5 Fig).

### In silico-predicted *P*. *litchii* pathogenesis-related proteins

Like other plant oomycete pathogens, *P*. *litchii* presumably secretes a battery of virulence proteins to promote infection. A total of 978 secrete proteins were identified in *P*. *litchii* using Signal[41]. Pathogenicity-related proteins (except for RXLR and CRN) were identified by comparison of *P*. *litchii* genes against public databases. A total of 490 pathogenicity-related genes with known or putative roles in virulence were identified (Table 3). Compared with two other oomycete species (*P*. *sojae* and *H*. *arabidopsidis*), the families of glycosyl hydrolases were significantly reduced in *P*. *litchii* (Table 3), whereas the families encoding ABC transporters were expanded (Table 3). The *P*. *litchii* families encoding host target, degradative enzymes (polygalacturonases, pectin methylesterases, pectinesterases, and extracellular proteases) and elicitin-like proteins were about half of these in *P*. *sojae* and more than two-fold of *H*. *arabidopsidis* (Table 3). Results further revealed that *P*. *litchii* contained relatively fewer genes involved in hydrolytic enzymes than *P*. *sojae*. Protease inhibitors and NPP1-like proteins, which have been implicated in the transition from biotrophy to necrotrophy were abundant in *P*. *litchii* and *P*. *sojae* (Table 3). Other apoplastic effectors groups including CBEL-like proteins, crinklers, and RXLR proteins had a similar level in *P*. *litchii* and *H*. *arabidopsidis* (Table 3).

**Table 3:** Gene families of *P*. *litchii* potentially implicated in plant pathogenesis.

Gene families	*H*. *arabidopsidis*^a^	*P*. *litchii*	*P*. *sojae*^*a*^
ABC transporters, all	55	165	134
Glycosyl hydrolases	>60	27	125
Polygalacturonases	3	12	25
Pectin methylesterases	3	7	19
Pectinesterases	3	10	19
Extracellular proteases^b^	18	30	47
Elicitins and Elicitin-like	15	35	57
CBEL and CBEL-like	2	4	13
NPP1-like	10	29	29
Protease inhibitors, all	3	21	22
Crinklers	20	22	40
RXLR	134	128	396

^a^ Data from other oomycete species are from Tyler *et al*. for *P*. *sojae* and Baxter *et al*. for *H*. *arabidopsidis*. Counts of annotated pseudogenes are omitted.

^b^ Extracellular proteases refer to proteases with signal peptide within N-terminate.

Recent studies have shown a vast repertoire of cytoplasmic effector proteins, including RXLR and CRN families. The *P*. *litchii* gene dataset was searched against known oomycete effectors. As a result, 74 and 70 potential RXLR effectors were identified by blastx and hmmsearch, respectively. String searches for the motif within secretome yielded six additional potential RXLR effectors by Regex method. Finally, a total of 128 genes were identified as potential RXLR effectors (S2 Table). Furthermore, 22 genes were ascertained as RXLR effectors by all three methods (S2 Table). Among the 128 potential effectors, 87 candidates contained RXLR and/or dEER motif (S2 Table). The remaining 31 RXLR effector candidates contain either RXLR or dEER motifs (S2 Table), which may due to short sequences. Fifty genes both with signal peptide and RXLR motif were identified in potential RXLR effectors by using SignalP (S2 Table). WY-domain was another structure unit conserved and found in tandem repeats in many effectors. A total of 48 out of 128 (37.5%) potential RXLR effectors contained WY-domain, and 36 candidate effectors harbor more than one WY-domains (S2 Table). Furthermore, 14 out of 22 (63.6%) ascertained RXLR had WY-domains. Therefore, many RXLR genes of *P*. *litchii* comprised WY-domains (S2 Table). We performed similar searches to identify CRN effectors in *P*. *litchii*. In total, 22 potential CRN proteins with “LXLFLAK” motif were identified (Table 3).

### Phylogenetic analysis of *P*. *litchii*

**Fig 2:**
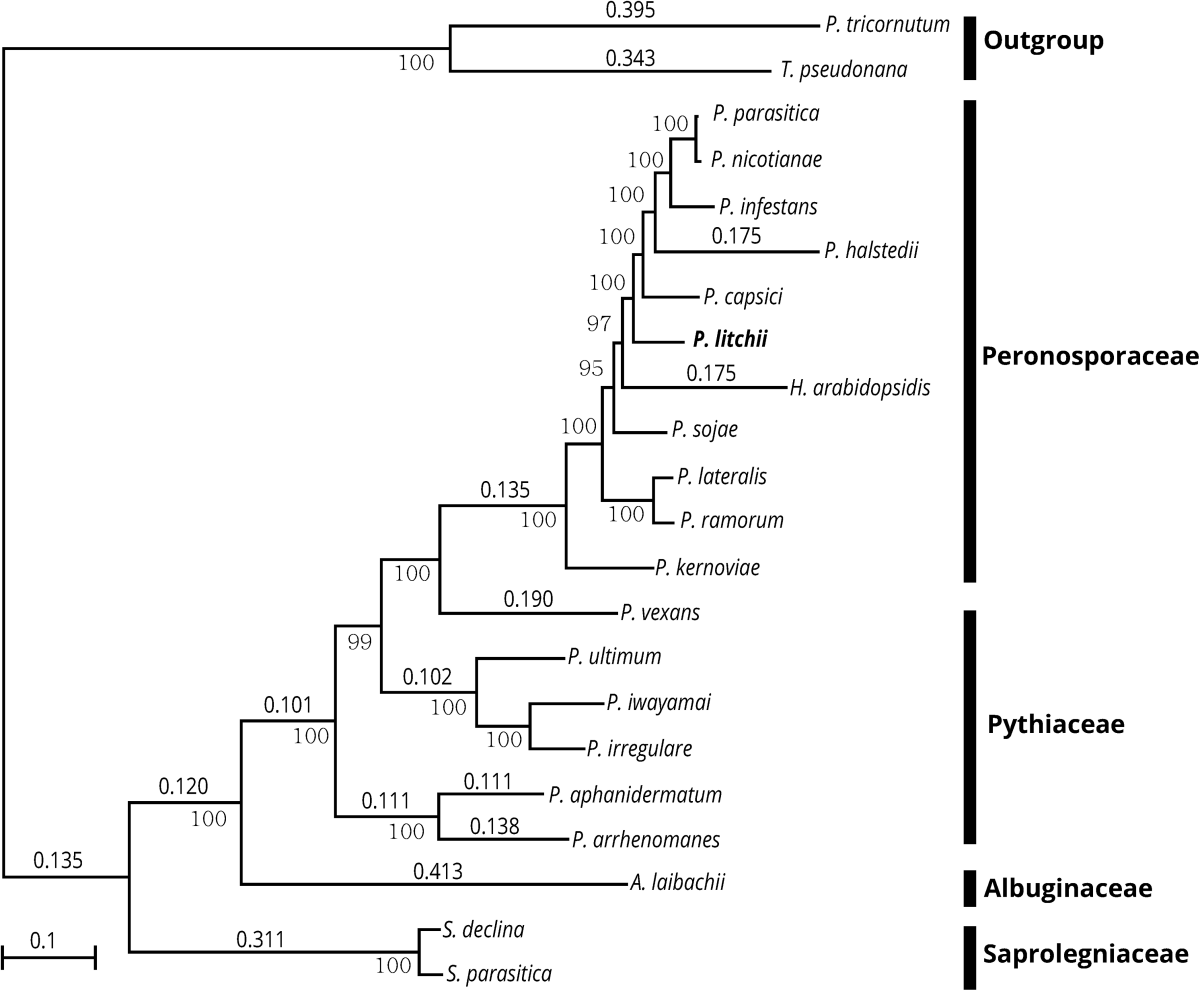
Phylogenetic tree of 22 oomycete species. Phylogenetic tree infered from the 164 single-copy proteins dataset by RAxML under the GAMMALGF model. ML bootstrap support (lower value) and genetic distance (upper value) were estimated under the uniform model.

The accuracy of species-tree estimates highly depends on the quality of initial phylogenetic data, usually gene trees, and multiple alignments in selected cases. Due to rapid gene evolution, gene recombination, and horizontal gene transfer, the trees constructed from different genes often were conflicting and resulted in an incorrect species tree. To reconstruct a reliable species phylogeny, the multiple families of single-copy orthologs were identified among 22 species (Fig 2). We concatenated alignments of 164 single-copy orthologs (included 143458 amino acid positions) and inferred the species tree using a maximum likelihood approach[43]. The obtained species phylogeny was highly supported with bootstrap values more than 90% for all nodes. With *P*. *tricornutum* and *T*. *pseudonana* set as outgroup, the branch of Saprolegniaceae (*S*. *declina* and *S*. *parasitica*) was off firstly and followed by the branch of Albuginaceae. After the six *Pythium* species were off in the evolutionary tree, the eleven members of Peronosporaceae clustered into a group (Fig 2). *P*. *litchii* was the neighbor of *P*. *capsica* and *H*. *arabidopsidis*(Fig 2). Two species of downy mildews (*H*. *arabidopsidis* and *P*. *halstedii*) were nested within *Phytophthora* clade with high bootstrap support and were separated by *P*. *litchii* and *P*. *capsici*. The branch length of *P*. *halstedii* and *H*. *arabidopsidis* both represented 0.175 genetic distance and were much longer than other nine members of Peronosporaceae (Fig 2).

## Discussion

Despite the severe economic pre- and post-harvest losses caused by *P*. *litchii*, little is known about the genetic information and molecular basis of its pathogenicity. The availability of genetic sequences from other pathogens has greatly advanced the understanding of this pathogen. A lag still exists in developing these resources for *P*. *litchii*. High-throughput sequencing is a powerful and economical technology to uncover new genes and their involved biochemical pathways in non-model species[47–50]. Transcriptome analysis provide abundant sequence information and shed light on the molecular mechanism of pathogenicity. In the present work, 23637 transcripts of *P*. *litchii* were assembled. The average length (1284 bp) of transcripts was longer than that (1116 bp) of *Phytophthora cactorum* obtained using a similar approach[49]. The gene number of *P*. *litchii* transcriptome was comparable to that of *P*. *infestans*[10], but less than that of *P*. *sojae*[45] and more than that of *H*. *arabidopsidis*[11] and *P*. *halstedii*[18]. There were 13155 gene models in the genome assembly of *P*. *litchii* [17]. The total number of *P*. *litchii* genes was probably underestimated in genome project. It could be more accurately predicted if the transcriptome was incorporated into genome annotation. In addition, 97.2% of conserved core eukaryotic genes were mapped against the *P*. *litchii* transcriptome. The high mapped rate was comparable to that (93.6%) in *A*. *laibachii* and (95.0%) in *H*. *arabidopsidis* genome assemblies[11,51]. The similar result was displayed by BUSCO analysis. The high rate of identified CEGMA and BUSCO genes suggested a good assembly of *P*. *litchii*. Our work provided a high-quality transcriptome, which could be advantageous for further gene function validation and functional genomics approaches.

The conserved genes globally were examined by OrthoMCL across other four oomycete species. A total of 4666 gene families shared by all five species could be defined as the core gene set of Peronosporomycetidae (Fig 1). On the contrary, these genes lacked homologs in other species represented potentially *P*. *litchii*-specific members. Based on gene annotations and pathway analyses, the unigenes of *P*. *litchii* were predominantly involved in cellular and metabolic processes, and signal transduction. In addition, some important pathways such as “MAPK signaling pathway”, “calcium signaling pathway”, and “plant hormone signal transduction”, were identified and potentially involved in pathogenesis. The predicted pathways together with the gene annotations can be used to further investigate gene function in the future.

The transcriptome of *P*. *litchii* contained mass of genes encoding proteins that were potentially involved in pathogenicity. ABC transporters, which are found in all species and transport substrates across cellular membranes, is essential in cell viability, virulence, and pathogenicity in oomycetes. Compared to *P*. *sojae* and *H*.*arabidopsidis*, the expansion of ABC transporters was observed in *P*. *litchii*. It was implied that the transport activity between host and pathogen was more frequent than others. The families of glycosyl hydrolases were significantly reduced. Meanwhile, the families encoding degradative enzymes, polygalacturonases, pectin methylesterases, pectinesterases, and extracellular proteases, were less than those in *P*. *sojae* and more than those in *H*. *arabidopsidis*. In general, *P*. *litchii* contained a relatively fewer genes involved hydrolytic enzymes than *P*. *sojae*, in accordance with the fact that the fruit of litchi has a gap-peel structure and is penetrated through the crack of pericarp by *P*. *litchii*. Thus, it is not necessary to maintain mass hydrolases for cell wall collapse.

The species of *Phytophthora* and downy mildews secrete a vast repertoire of effector proteins that modulate host defenses and enable pathogenicity[10,19,29]. In the present study, we examined the similarity to known oomycete effectors. NPP1-like proteins have been implicated in the transition from biotrophy to necrotrophy. Compared to 30 NPP1-like proteins in assembly of genome, there were 29 members identified in transcriptome of *P*. *litchii*. These genes were abundant in *P*. *sojae* and *P*. *litchii*. The NPP1-like proteins may have contributed to necrotrophy life style of *P*. *litchii*. CBEL-like proteins, another apoplastic effectors, were also detected but had a similar level in *P*. *litchii* and *H*. *arabidopsidis* (Table 3). In addition to apoplastic effectors, there were also a large repertoire of candidate CRN effectors that elicit crinkling and necrosis in planta. The transcriptome of *P*. *litchii* annotated 22 candidate CRN effectors, which were little more than 14 members in assembly of genome. The number discrepancies between the assembly of genome and transcriptome were most likely the result of incomplete assembly of genome or chimeric assembly of transcriptome. We identified 128 potential RXLR effectors in the *P*. *litchii* transcriptome. The C-terminal regions of RXLR effectors fulfill the effector activity. About half of them share a conserved WY-domain, which is an adaptive structure[31]. Using HMM-based method, WY-domains were also detected in our potential RXLR effectors, 37.5% of which contained the WY-domain. These candidate RXLR effectors contained sequence features and motifs characteristic of the canonical RXLR effectors. There were 245 candidate RXLR effectors in genome annotation[17]. The transcripts of *P*. *litchii* represented these genes expressed during culture but did not represent all genes of genome. The effector data set could be expanded by including additional life cycle and infection stages of *P*. *litchii*. Our data indicated that *P*. *litchii* exhibited the same kind of pathogenicity-related proteins found in other oomycetes.

The relationship of *Phytophthora*, *Peronophythora*, and downy mildews is still under debate. *Phytophthora* and *Peronophythora* can grow on artificial media, whereas downy mildews cannot be culture under in vitro conditions. Meanwhile, *Peronophythora* and downy mildews are determinate and produce differentiated sporangiophores. *Peronophythora* is a genus with characteristics intermediate between downy mildews and *Phytophthora*[2,3]. *Phytophthora* and downy mildews share an intimate relationship. These are both morphologically and phylogenetically connected by “bridging taxa” of the graminicolous downy mildews such as *Viennotia*, *Poakatesthia*, and *Sclerophthora*[52]. The traditional classification has the limitation of the taxonomy-based-morphology. These methods quickly lose power when too many species are included or when dealing with specimens whose closest phylogenetic relatives are unknown. Molecular data, especially DNA sequence, can be used for the rapid identification and delimitation of species. Through species classification analysis, *P*. *litchii* and *P*. *sojae* shared the highest level of similarity across all NCBI Nr databases (S2 Fig). Meanwhile, *P*. *litchii* shared more specific gene families with *P*. *sojae* than *H*. *Arabidopsis*. These results implied that *P*. *litchii* was closer to *Phytophthora* than downy mildews. Recently studies have suggested that the three genera are included in one monophyly, in which *Phytophthora* is paraphyletic and downy mildews is nested within clade 4 of *Phytophthora*[15]. *P*. *litchii* is also included into clade 4 of *Phytophthora*[15]. Previous studies on *P*. *litchii* phylogenies have focused primarily on 28S and ITS sequences. In phylogenetic analyses, different single-gene data sets have resulted in controversial phylogenies in certain cases even within the same species group. But large numbers of characters and independent evidence from many genetic loci often result in well-resolved and highly supported phylogenetic hypotheses. For these reasons, the analysis of multi-gene sequence data is becoming increasingly common. 164 single-copy orthologs were used to determine the taxonomic position of *P*. *litchii* and the relationship of *Phytophthora* and downy mildews. The obtained species phylogeny was supported with high bootstrap values (>94) for all nodes (Fig 2). Phylogenetic analysis revealed a closer relationship between *P*. *litchii* and *P*. *capsici* (Fig 2). *P*. *capsici* falls within clade 2 of *Phytophthora*, which is neighbor to clade 4[15]. It is confirmed that *P*. *litchii* is a member of *Phytophthora*. However, two downy mildews, *P*. *halstedii* and *H*. *arabidopsidis*, were separated by *P*. *litchii* and *P*. *capsica*. The results refuted monophyly of the downy mildews and were consistent with the phylogenies analysis in recently studies[18]. The longer branch length of two downy mildews was obvious. It indicated that the two of downy mildews had more divergent mutation rates than nine *Phytophthora* species (Fig 2). It maybe is a result of biotrophic life in downy mildews. It should be noted that *P*. *litchii* was adjacent to *H*. *arabidopsidis* in genetic levels. Considering the character of sporanigia, *P*. *litchii* can be classified as bridging species between *Phytophthora* and downy mildews. In summary, the molecular evidence presented in this work confirms that *P*. *litchii* belong to *Phytophthora* and is a bridging species between *Phytophthora* and downy mildews.

## Supporting information

S1 Table The KEGG pathway classfication of *P*. *litchii*. (XLSX)

S2 Table Canonical and variant RXLR-dEER motifs and the WY-domain found in transcriptome of *P*. *litchii*. (XLSX)

S1 Fig Length distribution of *P*. *litchii* transcriptome. (TIFF)

S2 Fig Species classification of *P*. *litchii*. (TIFF)

S3 Fig Functional annotation of *P*. *litchii* transcriptome based on GO categories. The results are summarized in three main categories: biological process, cellular component and molecular function. A set of 17647 genes were assigned to GO term based on blastx matches to known proteins.
(TIFF)

S4 Fig Histogram of KOG classification of *P*. *litchii* genes. In total, 6920 of the 19627 *P*. *litchii* genes with Nr hits were grouped into 25 KOG classification.
(TIFF)

S5 Fig KEGG pathway annotation of *P*. *litchii* transcriptome. Pathway assignment was summarized for five main categories: Cellular Processes (C), Environmental Information Processing (E), Genetic Information Processing (G), Metabolism (M) and Organismal Systems (O). A total of 4624 genes were assigned to 32 groups, and abundant genes were involved signal transduction.
(TIFF)
