# Supplementary figures and images for: Transcriptome analysis of Phytophthora litchii reveals pathogenicity arsenals and confirms taxonomic status
Source: PLoS One. 2017 Jun 1;12(6):e0178245. doi: 10.1371/journal.pone.0178245 (PMC5453482; doi:10.1371/journal.pone.0178245)

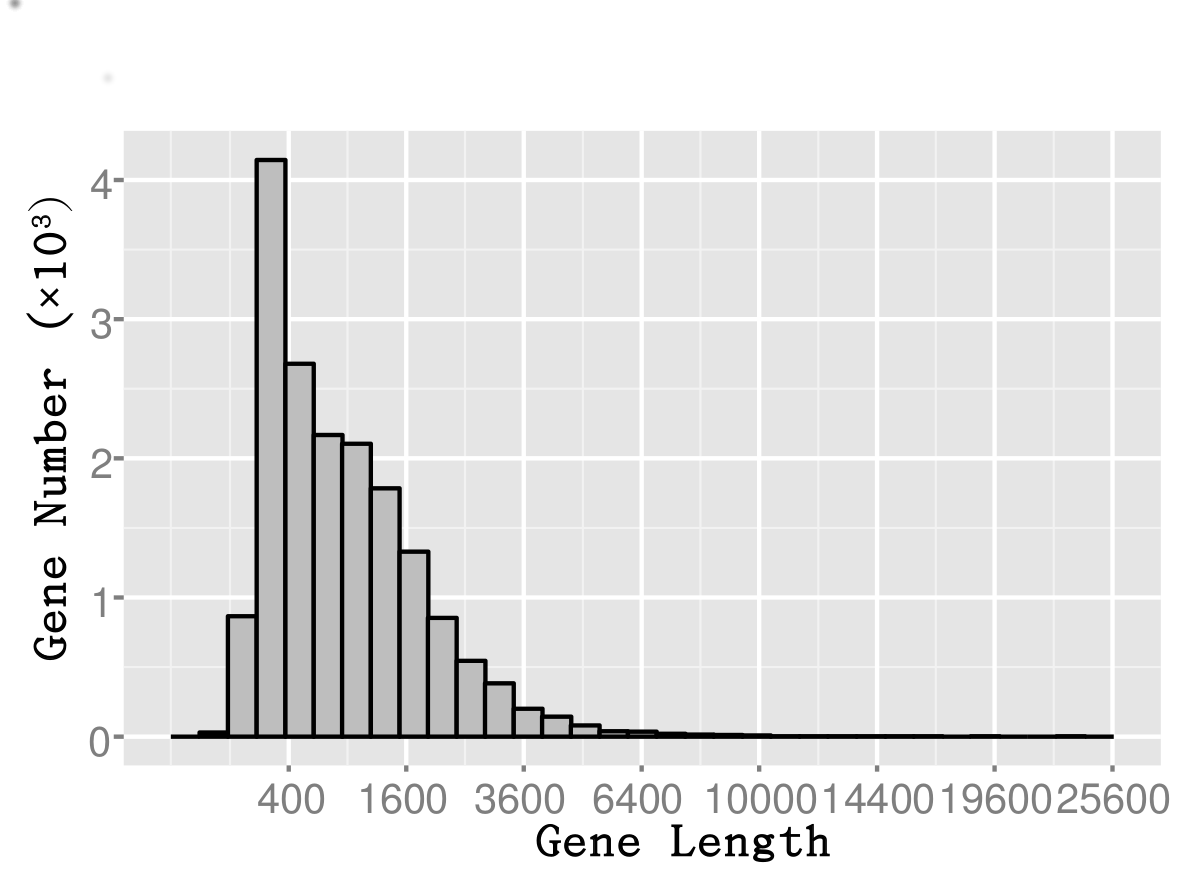

Supplement: S1 Fig — (TIFF) [file pone.0178245.s003.tiff]

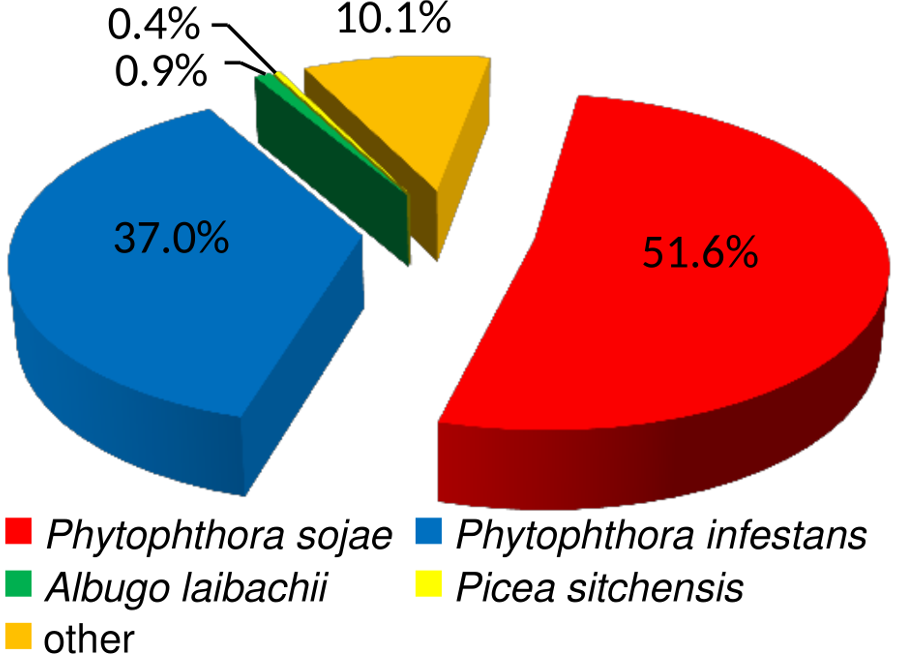

Supplement: S2 Fig — (TIFF) [file pone.0178245.s004.tiff]

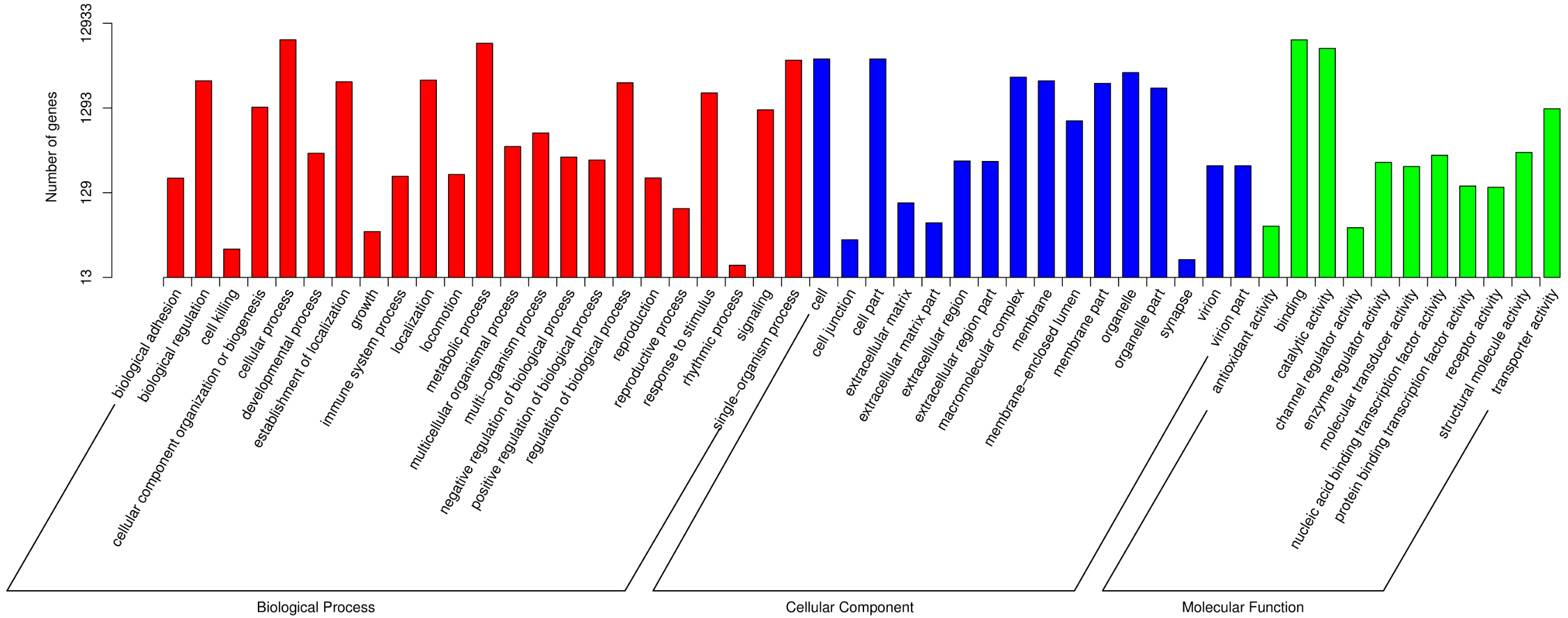

Supplement: S3 Fig — The results are summarized in three main categories: biological process, cellular component and molecular function. A set of 17647 genes were assigned to GO term based on blastx matches to known proteins. (TIFF) [file pone.0178245.s005.tiff]

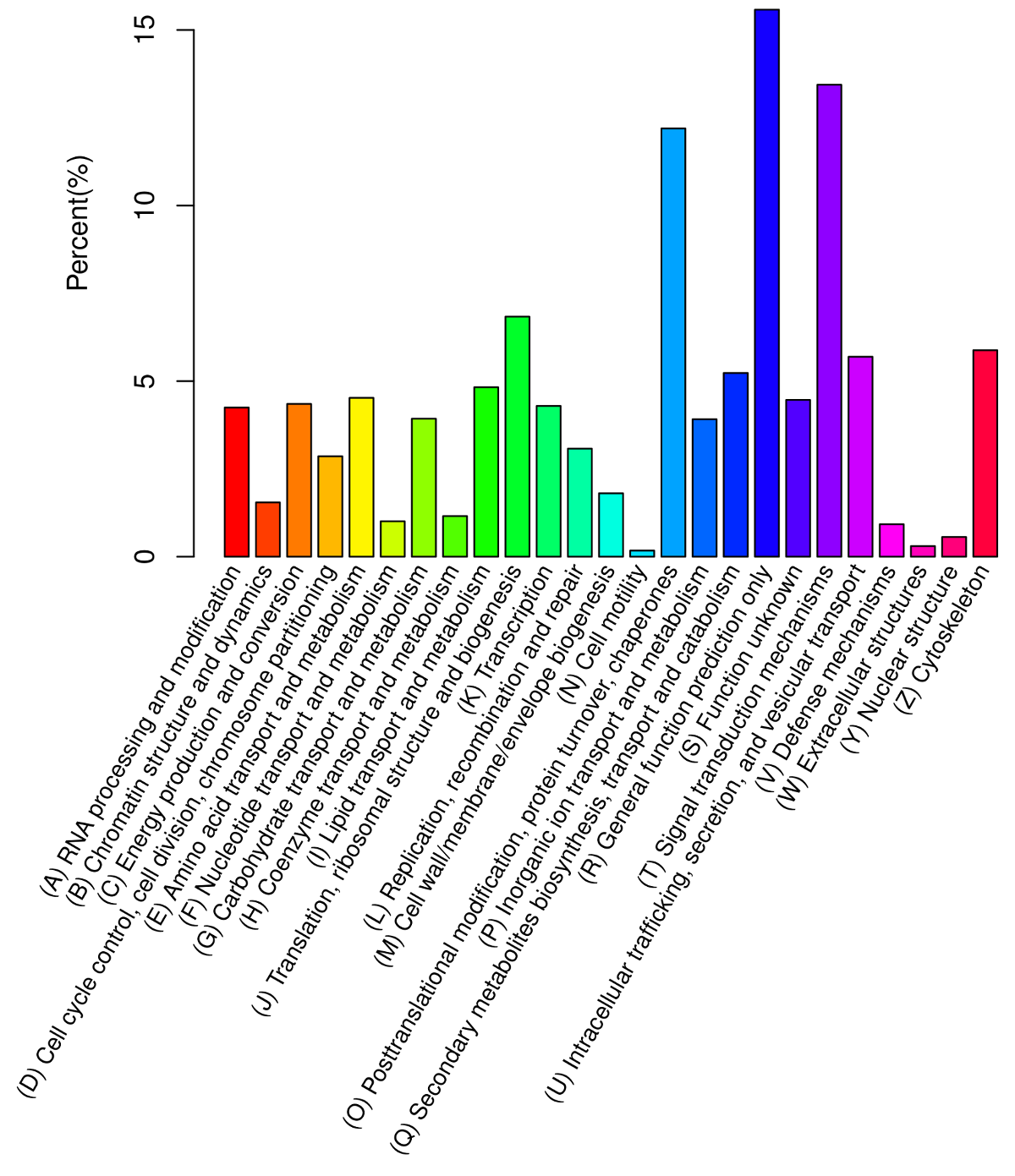

Supplement: S4 Fig — In total, 6920 of the 19627 P. litchii genes with Nr hits were grouped into 25 KOG classification. (TIFF) [file pone.0178245.s006.tiff]

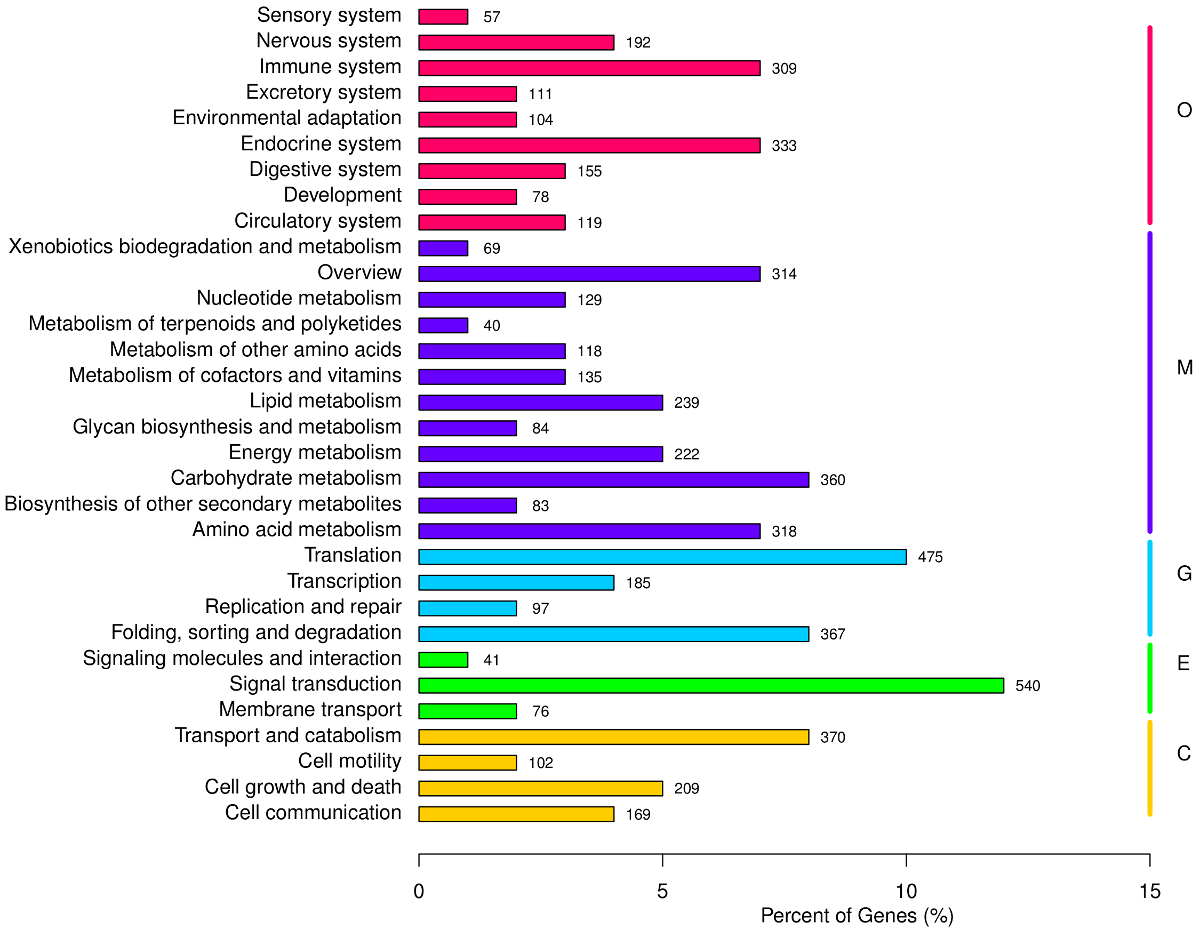

Supplement: S5 Fig — Pathway assignment was summarized for five main categories: Cellular Processes (C), Environmental Information Processing (E), Genetic Information Processing (G), Metabolism (M) and Organismal Systems (O). A total of 4624 genes were assigned to 32 groups, and abundant genes were involved signal transduction. (TIFF) [file pone.0178245.s007.tiff]
